# Editorial: Innovation in ocular pharmacology

**DOI:** 10.3389/fphar.2023.1242014

**Published:** 2023-10-05

**Authors:** Lucia Gozzo, Mario Damiano Toro, Vittorio Porciatti, Giovanni Luca Romano

**Affiliations:** ^1^ Clinical Pharmacology Unit/Regional Pharmacovigilance Centre, Azienda Ospedaliero Universitaria Policli-nico “G. Rodolico—S. Marco”, Catania, Italy; ^2^ Eye Clinic, Public Health Department, University of Naples Federico II, Naples, Italy; ^3^ Bascom Palmer Eye Institute, Miller School of Medicine, University of Miami, Miami, FL, United States; ^4^ Department of Biomedical and Biotechnological Sciences, University of Catania, Catania, Italy

**Keywords:** innovative treatment, pharmacological targets, ocular pharmacology, unmet medical need, ocular disease

An innovative medicinal product can be a new active substance acting against or preventing a disease and developed to improve the quality of patient management.

Thus, product novelty alone is not sufficient to characterize therapeutic innovation, as an improvement of health outcomes is necessary.

Recognizing a true innovation can accelerate the development and adoption of valuable treatment options, encouraging their prioritization to make them quickly available for patients with high unmet medical needs.

This Research Topic collects updated evidence about *Innovation in ocular pharmacology*, a field which rapidly grown over the last few years, leading to the discovery of disease modifying treatments for several eye disorders.

Nevertheless, several unmet needs remain, such as retinal diseases (Cursiefen et al., 2019; Conti et al., 2021; Kaminska et al., 2021), characterized by a devastating impact on the quality of life but also on the healthcare system and the society.

Anti-vascular endothelial growth factor (anti-VEGF) agents represent the mainstay of treatment for neovascular age-related macular degeneration (AMD) (Jonas et al., 2017). However, this therapy can slow the progression but do not cure the disease.

Abdin et al. analysed data from the first year of treatment with brolucizumab for refractory neovascular AMD. This study showed that the drug allows to stabilize visual acuity with less injections in patients with refractory disease, to reduce subretinal fluid and pigment epithelial detachment.

Moreover, the intraocular injection is associated with the risk of several complications, including inflammation, intraocular pressure elevation, hemorrhage, and endophthalmitis, which can lead to significant visual loss (Falavarjani and Nguyen, 2013).

In this regard, Dolar-Szczasny et al. evaluated the onset of ocular inflammation after repeated bevacizumab injections in patients with AMD. The study showed that none of the subjects treated with bevacizumab had detectable inflammation during follow-up, suggesting a good safety profile of the drug.

On the contrary, no specific treatments are currently available for dry AMD. The study of Melecchi et al. compared the efficacy of an oral formulation based on lutein and fish oil, as a source of omega-3, with a combination of lutein and astaxanthin with Calanus oil (COil), containing omega-3 and their precursors policosanols in a mouse model of dry AMD. Both mixtures demonstrated to exert a significant antioxidant and anti-inflammatory activity, in particular the formulation based on COil. These results support the use of dietary supplements in the prevention and treatment of AMD, suggesting the potential role of fatty acids of COil origin as AMD modifying therapies with higher efficacy compared to fatty acids of fish oil origin.

Anti-VEGF monotherapy represents the first line treatment for Retinal Angiomatous Proliferation (RAP), a form of neovascular AMD. RAP lesions may be difficult to treat and may have a worse response compared with other forms of AMD. In this regard Fallico et al. compared the functional and anatomical outcomes of anti-VEGF monotherapy with the combination of anti-VEGF and Photodynamic Therapy (PDT) in a systematic review with meta-analysis. The study showed that a combined approach with anti-VEGF and PDT could provide better functional and anatomical outcomes in RAP compared with monotherapy.

Diabetic retinopathy (DR) still represents a major cause of impaired vision and blindness. In these patients, oxidative stress, inflammation, and vascular dysfunction lead to retinal ischemia and blood retinal barrier (BRB) impairment (Bucolo and Drago, 2004). Vitamin D3 have been studied in several eye diseases, for its anti-inflammatory, antioxidant and anti-angiogenic activity. The aim of the study of Lazzara et al. was to assess the effects of vitamin D3 on BRB damage using human retinal endothelial cells exposed to high glucose levels. Vitamin D3 demonstrated to preserve the BRB integrity, attenuating cell damage, and reducing the level of inflammatory cytokines. This study supports the protective role of vitamin D3 in DR, characterized by inflammation and BRB disfunction, but also in other retinal conditions.

Diabetic macular edema (DME) is the major cause of vision deterioration in patients with DR. The dexamethasone (DEX) intravitreal implant is a biodegradable device which slowly release DEX for up to 6 months, approved for the management of DME (Mathis et al., 2020). Several studies compared the safety and effectiveness of the DEX implant for DME in nonvitrectomized and vitrectomized eyes. Yuan et al. conducted a systematic review and meta-analysis to compare the improvements of DME with DEX implant in nonvitrectomized and vitrectomized eyes. The study showed no significant differences in terms of anatomical and functional effects between vitrectomized and nonvitrectomized eyes, with good safety profile.

Zerbini et al. focused on the possibility to prevent DR using topical nerve growth factor (NGF). The study showed that retinal neurodegeneration represents an early self-limiting phenomenon in the first stage of the disease, followed by vascular dysfunctions. Topical administration of NGF demonstrated to prevent neurodegeneration, but also the development of the vascular damage of DR.

Retinopathy of prematurity (ROP) is a primary cause of blindness in children characterized by abnormal retinal vessel development (Alajbegovic-Halimic et al., 2015). Currently, laser photocoagulation and anti-VEGF agents are the first- and second-line therapies to treat ROP, but they are invasive methods, and no long-term data are yet available (Shulman and Hartnett, 2018; Stahl et al., 2019). RNA therapy has been investigated in several diseases and showed potential as innovative therapy in ophthalmology. Kim et al. reviewed available evidence about noncoding RNAs (ncRNAs) as treatment for ROP.

Finally, two papers focused on ocular surface diseases. Gao et al. compared the effectiveness and safety of different formulations of cyclosporine A (CsA) for the treatment of dry eye disease using a network meta-analysis including high-quality placebo-controlled trials. The results of the study suggest that various formulations of CsA are effective in the treatment of dry eye, but it’s difficult to select the optimal one.

After a corneal damage, consequent to various insults including dry eye disease, it is essential to restore the epithelium, the stroma, but also the nervous components. Bucolo et al. performed *in vivo* and *in vitro* studies with six different ocular formulations to evaluate corneal nerve regeneration and corneal wound healing after corneal damage. A new ophthalmic gel containing cross-linked sodium hyaluronate, taurine, vitamin B6 and Vitamin B12 demonstrated to better reduce oxidative stress, to accelerate corneal re-epithelization and to promote nerve regeneration.
